# Factors affecting mortality after coronary bypass surgery: a scoping review

**DOI:** 10.1186/s13019-022-01784-z

**Published:** 2022-03-21

**Authors:** Sean Christopher Hardiman, Yuri Fabiola Villan Villan, Jillian Michelle Conway, Katie Jane Sheehan, Boris Sobolev

**Affiliations:** 1 School of Population and Public Health, University of British Columbia, 828 West 10th Avenue, Vancouver, BC V5Z 1M9 Canada; 2 Hospital Universitario La Paz, Madrid, Spain; 3 Faculty of Medicine, University of Ottawa, Ottawa, Canada; 4 Department of Population Health Sciences, School of Population Health and Environmental Sciences, King’s College London, London, UK

**Keywords:** Scoping review, Coronary artery bypass graft, Mortality

## Abstract

**Objectives:**

Previous research reports numerous factors of post-operative mortality in patients undergoing isolated coronary artery bypass graft surgery. However, this evidence has not been mapped to the conceptual framework of care improvement. Without such mapping, interventions designed to improve care quality remain unfounded.

**Methods:**

We identified reported factors of in-hospital mortality post isolated coronary artery bypass graft surgery in adults over the age of 19, published in English between January 1, 2000 and December 31, 2019, indexed in PubMed, CINAHL, and EMBASE. We grouped factors and their underlying mechanism for association with in-hospital mortality according to the augmented Donabedian framework for quality of care.

**Results:**

We selected 52 factors reported in 83 articles and mapped them by case-mix, structure, process, and intermediary outcomes. The most reported factors were related to case-mix (characteristics of patients, their disease, and their preoperative health status) (37 articles, 27 factors). Factors related to care processes (27 articles, 12 factors) and structures (11 articles, 6 factors) were reported less frequently; most proposed mechanisms for their mortality effects.

**Conclusions:**

Few papers reported on factors of in-hospital mortality related to structures and processes of care, where intervention for care quality improvement is possible. Therefore, there is limited evidence to support quality improvement efforts that will reduce variation in mortality after coronary artery bypass graft surgery.

**Supplementary Information:**

The online version contains supplementary material available at 10.1186/s13019-022-01784-z.

## Strengths and limitations of this study

- Comprehensive sample from 2000 to 2019 included.
- Only articles with well-defined study groups included.
- Mechanisms for associations extracted.
- Scoping review according to PRISMA-ScR guidelines.

## Introduction

Coronary artery bypass grafting (CABG) is a safe treatment for patients with coronary artery disease [1]. Progressive improvements in post-operative survival over its fifty-year history have been observed through improvements in surgical technique [2, 3]. Canadian reports estimate 30-day mortality after isolated CABG surgery is 1.3% [4]. However, regional variation in mortality has been observed in Canada [4, 5], the United States [6, 7], and the United Kingdom [8]. Previous research reported numerous factors contributing to this variation. Indeed, fewer deaths are reported in patients with favorable case mix characteristics, including those who are younger [9], have normal ejection fractions [10] and are free of iron deficiency [11]. Care-related factors, including higher hospital and surgeon volume [12, 13], and process factors, such as the use of arterial grafting strategies [14], are also shown to contribute to lower mortality.

However, the entirety of this evidence has not been mapped on the conceptual framework of quality improvement. Without such mapping, designing interventions to improve care quality could be misguided [15].

Many quality improvement initiatives use the Donabedian framework [16] that considers factors related to structures and processes of care. In this framework, structures include the care provider organizational features (services, size, systems, and volume), the human resources (experience and qualifications) and the material resources (equipment, facilities, and staffing ratios) required to provide care. Processes refer to managerial activities (prioritization, scheduling, and discharge planning) and the medical procedures (both diagnostic and treatment) that constitute care delivery within the defined structures. Outcomes refer to the results that may stem from exposure to a factor; in this report we refer to intermediary outcomes to identify factors that occur after exposure to CABG but prior to in-hospital mortality as the terminal outcome. Shroyer et al. [17] augmented this framework by including factors related to patients and disease, which we refer to as ‘case-mix’ in this report.

Scoping reviews use a systematic approach to map evidence on a topic and identify main concepts, as well as knowledge gaps [18]. We use the scoping review methodology to select factors of in-hospital mortality for patients undergoing isolated CABG and map them to the augmented Donabedian framework. We then synthesize information on mechanisms for their effects.

## Methods

This review adheres to the Scoping Review extension of the Preferred Reporting Items for Systematic Review and Meta-Analysis statement (PRISMA-ScR) [18].

### Eligibility criteria

We included observational studies which reported the association from the regression analysis of postoperative in-hospital mortality among patients aged 19 years and older who underwent isolated CABG, published in English between January 1, 2000 and December 31, 2019 (Table 1).

**Table 1:** Selection criteria for the literature search

Term	Include
Study population	Men and women ≥ 19 years of age who underwent isolated CABG
Study design	Observational studies
Factors	Patient, structures, or processes of perioperative care
Associations	Estimates from regression analysis
Outcome	In-hospital mortality
Date	Between January 1, 2000 and December 31, 2019
Language	English
Geography	Worldwide

We defined risk factors as any attribute, characteristic, or exposure that increases the likelihood of developing a disease or incurring an injury [19]. We excluded intervention studies, any composite outcome of complications and mortality, a study endpoint outside of the hospital setting, and studies where no statistical association was found.

### Information sources and search strategy

We searched the electronic databases PubMed, CINAHL, and EMBASE for studies published between 2000 and 2019. Reference lists of retrieved studies were further screened to identify additional studies that may have been missed during database searches.

### Search

The search was developed using terms for the intervention (coronary artery bypass graft), outcome (mortality), study design (observational), and analysis (regression) (see Additional file 1 for full electronic search strategy for each database).

### Selection of sources of evidence

We exported citations from databases into reference management software for de-duplication prior to screening. Two reviewers independently screened all abstracts against inclusion and exclusion criteria. Conflicts were resolved by consensus. Full texts of potentially eligible studies were independently screened by two reviewers with conflicts resolved by consensus.

### Data chart processing and data items

We used a pre-designed form to collect data; the form was piloted by two reviewers on five articles. No conflicts in data extraction were noted. Data extracted included the author’s name, publication date, country, study population, study design, sample size, source of data, risk factor measurement, outcome, and effect estimate. We identified risk factors representing the exposure, treatment, or intervention of primary interest in the title or objectives of the selected papers. We extracted the effect of the primary risk factor from multivariable analysis from papers with well-defined study groups [20]. This was done to avoid misclassification of covariates in multivariable analyses as primary factors [21]. Factors were considered statistically significant when a *p*-value < 0.05 was reported. The proposed mechanisms for reported associations were extracted from the discussion section by one reviewer. The extraction was checked for accuracy by a second reviewer.

### Synthesis of results

We summarized the data in text, tables, and figures. Two authors sorted factors according to common properties, creating 14 unique groups. Two authors then mapped the groups to case-mix, structures, processes, and intermediary outcomes of the augmented Donabedian framework. Disagreements on sorting, grouping, and labelling were resolved by consensus. Finally, we synthesized proposed mechanisms, where mechanisms were reported, for the association between factors and in-hospital mortality from articles in which they were identified (Table 3).

### Patient and public involvement

Patients were not involved in the design, conduct, reporting, or dissemination plans of our research.

## Results

### Search results

**Fig. 1:**
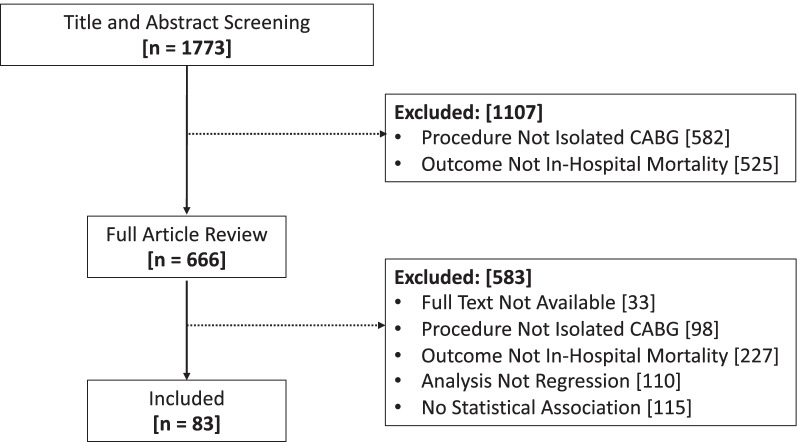
Flow chart of the literature, retrieval, review, exclusion, and selection process

The search produced 1773 articles for initial title and abstract screening (Fig. 1). We excluded 1107 articles on title and abstract screening: 582 were not isolated CABG; 525 were not in-hospital mortality. We further excluded 583 articles on full-text screening: 33 had no full-text available, 98 were not isolated CABG, 227 had an outcome that was not in-hospital mortality, 110 used an analysis that was not multivariable regression, and 115 had results where there was no statistical association found. 83 articles remained to be included in the review.

### Structural factors of in-hospital mortality

In total, 12 articles reported on structural factors of in-hospital mortality after CABG. Factors in these articles were grouped to treatment era [earlier year of operation (n = 4)], care setting [hospital volume (n = 4), hospital type (n = 1)], and operator qualification [operator volume (n = 2), surgeon experience (n = 1)] (Table 2). Of the 6 identified factors, we synthesized 4 mechanisms from 7 articles for their effect on mortality (Table 3).

**Table 2:** Grouped factors of postoperative mortality in coronary bypass surgery by reviewed article

Author	Treatment era	Care setting	Operator qualification	Preoperative care	Intraoperative management	Postoperative care	Treatment delay	Complications	Sociodemographic factors	Health risks	Disease characteristics	Disease history	Comorbidity burden	Operative risk factors
Alam [22]									X					
Allareddy [23]		X												
Atalan [24]										X				
Azarfarin [25]													X	
Benedetto [26]		X	X											
Blankstein [27]									X					
Borgermann [28]					X									
Bybee [29]				X										
Chen [30]													X	
Chikwe [31]													X	
Connolly [32]									X					
Dacey [33]				X										
Dacey [34]														X
Davierwala [35]	X													
Engel [36]										X				
Foroughi [37]													X	
Furnary [38]				X										
Gan [39]				X										
Garcia-Fuster [40]														X
Glance [41]								X						
Gopaldas [42]	X													
Grieshaber [43]				X										
Grossi [44]					X									
Halkos [45]													X	
Hannan [46]								X						
He [47]					X									
Huffmyer [48]				X										
Keeling [49]										X				
Kim [50]		X												
Koch [51]					X									
Konety [52]					X									
Lahtinen [53]								X						
Lee [54]							X							
Li [55]					X									
Li [56]													X	
Liakopoulos [57]				X										
Lin [58]														X
Lin [59]				X										
Liu [60]													X	
Lopez-de-Andres [61]													X	
Mack [62]					X									
Magee [63]					X									
Magovern [64]				X										
Malaisrie [65]													X	
Mangano [66]				X										
Massoudy [67]												X		
McNeely [68]	X													
McNeil [69]											X			
Minakata [70]													X	
Mizutani [71]					X									
Mohnle [72]						X								
Monteiro [73]							X							
Najafi [74]													X	
Nallamothu [75]									X					
Paone [76]					X									
Pouleur [77]													X	
Pullan [78]					X									
Ramsey [79]					X									
Rathore [80]		X												
Rogers [81]					X									
Rosenthal [82]		X												
Schneeweiss [83]				X										
Sharony [84]					X									
Sharony [85]					X									
Sobolev [86]							X							
Sobolev [87]							X							
Swaminathan [88]									X					
Thielman [89]												X		
Thielman [90]												X		
Tooley [91]													X	
Toumpoulis [92]								X						
Wang [93]														X
Wang [94]														X
Wang [95]														X
Warwick [96]														X
Weerasinghe [97]														X
Weiss [98]							X							
Wen [99]			X											
Woods [100]									X					
Wu [101]			X											
Yanagawa [102]	X													
Zakeri [103]													X	
Zhong [104]													X

**Table 3:** Synthesized mechanisms proposed for case-mix characteristics, structures, processes, and intermediary outcomes in reviewed articles

	Group	Factor	Proposed mechanism
Structures	Treatment era	Earlier year of operation	Improved perioperative care, including surgical techniques, and the increased rate of complete revascularization, have reduced in-hospital mortality over time [35, 42, 68, 102]
	Care setting	Hospital type	Lower volume CABG programs are present at Veterans Affairs hospitals compared to private hospitals with higher volume [82]
		Hospital volume	Increased procedure volume drive better care processes [23]
	Operator qualification	Operator volume	An inverse volume-outcome relationship, selective referral, and differences in case-mix characteristics drive differences in mortality between low-volume and high-volume operators [99]
Processes	Preoperative care	ASA administration	ASA has an irreversible effect on platelets, decreasing production of Thromboxane A2, reducing graft occlusion [29, 33]
		Beta blocker administration	Beta blockade may reduce the incidence of myocardial ischemia, through attenuation of heart rate [59]
		Insulin infusion	Pre-operative insulin reverses metabolic deficiencies in diabetics through a direct reduction of hyperglycemia [38]
		Intra-aortic balloon pump	Pre-operative intra-aortic balloon pump reduces left ventricular afterload and increases coronary perfusion [43]
		Statin administration	Statins confer protection from the inflammatory response by reducing cytokine release and neutrophil adhesion, improving post-operative myocardial perfusion [39, 48, 57, 64]
	Intraoperative management	Allogenic blood transfusion	Leukocytes in allogenic blood cause widespread leukoreduction of blood components [81]
		Intra-aortic balloon pump	Intraoperative intra-aortic balloon pumps support circulation by reducing cardiac load and decreasing dependence on vasoactive medications [47]
		Off-pump cardiopulmonary bypass	Selection of off-pump cardiopulmonary (OPCAB) bypass is a function of the patient’s perioperative risk profile, including sex, comorbidities, extent of disease, and physician practice. OPCAB removes the systematic inflammatory response and complications associated with the use of cardiopulmonary bypass, possibly due to less aortic manipulation [28, 52, 55, 62, 71, 78, 84, 85]
		Pulmonary artery catheterization	Increased experience with pulmonary artery catheter insertion may affect in-hospital mortality [79]
		Red blood cell transfusion	Immunosuppressive and inflammatory effects, poor oxygen delivery, and red blood cell deformity may contribute to poorer outcomes [76]
	Postoperative care	Red blood cell transfusion	Transfusion may cause an increase in blood viscosity and shear forces with subsequent increases in platelet activation [72]
Intermediary Outcomes	Complications	Pulmonary artery temperature	Patients with warmer pulmonary artery temperatures are at higher risk of adverse events [53]
Case-mix characteristics	Sociodemographic factors	Medicaid insurance and uninsured status	The type of insurance affects access to preoperative care in the United States [32]
		Native American status	Diet and lifestyle behaviors increase the prevalence of diabetes [75]
		Sex	Females have lower body surface area, thought to correspond to smaller coronary artery size resulting in technical difficulties grafting to smaller targets and longer lifespan resulting in later CAD presentation [22, 27, 88, 100]
	Health risks	Body mass index	Obese patients have lower systemic vascular resistance and higher plasma renin activity, while patients who are underweight may have increased levels of inflammation which could lead to myocardial dysfunction [24]
	Disease history	Prior percutaneous coronary intervention [PCI]	PCI procedures cause inflammatory reactions leading to post-stenting endothelial injury and dysfunction. Intimal hyperplasia, along with platelet and neutrophil adhesion increase the risk of thrombosis [67, 89, 90]
	Comorbidity burden	Atrial fibrillation	Patients with AF have higher incidence of thromboembolic events and post-operative low cardiac output syndrome [65]
		Dialysis-dependent renal failure	Dialysis-dependent patients in renal failure may have a higher burden of atherosclerotic disease involving multiple organs, be immunocompromised, and have poorer myocardial function [31, 60]
		Metabolic syndrome	Multiple complex metabolic reactions may directly or indirectly impact myocardial function and increase mortality [30]
		QT Prolongation	Demographic, congenital, structural, electrophysiological, and endocrine factors, along with medication use, may contribute to QT prolongation [37]
		Peripheral vascular disease	Patients with PVD may be ineligible for intra-aortic balloon pump support due to calcified ascending aortas [70]
		Peritoneal dialysis	Peritoneal dialysis patients had more postoperative complications, including sternal wound infection, stroke, higher usage of intra-aortic balloon pumps and extra-corporeal life support, and may have increased complications for early reintroduction of PD post-operatively [56, 104]
		Right ventricular systolic dysfunction	Increased pulmonary pressure and myocardial ischemia may contribute to right ventricular systolic dysfunction [77]
	Operative risks	Cockcroft-Gault formula	Cockcroft-Gault formula for calculating glomerular filtration rate (GFR) includes more variables than the MDRD equation and therefore may be more predictive kidney disease leading to increased risk of mortality [58]
		Forced expiratory volume 1 (FEV1)	Tobacco use may lead to COPD resulting in impaired lung function, compromising outcomes [40]
		Left atrial expansion index	Hypoxic, ischemic, and hyperkalemic changes after CABG increase left atrial expansion and atrial fibrillation, increasing risk of death [94]
		Red cell distribution width	Nutritional deficiency and recent blood transfusion could lead to increased mortality [96]
		White blood cell count	Increased white blood cell count may be a sign of preoperative infection [34]

### Process factors of in-hospital mortality

In total, 27 articles reported on process factors of in-hospital mortality after CABG. Factors in these articles were grouped to pre-operative care [aprotinin (n = 1), ASA (n = 3), beta blockers (n = 1), insulin infusion (n = 1), intra-aortic balloon pump (n = 1), statin (n = 4)], intraoperative management [allogenic blood transfusion (n = 1), cardiopulmonary bypass strategy (n = 10), packed red blood cells transfusion (n = 1), intra-aortic balloon pump (n = 1), and pulmonary artery catheterization (n = 1)], and postoperative care [red blood cell transfusion (n = 1)] (Table 2). Of the 12 identified factors, we synthesized 11 mechanisms from 22 articles for their effect on mortality (Table 3).

### Intermediary outcomes of in-hospital mortality

In total, nine articles reported intermediary outcome of in-hospital mortality after CABG. Factors in these articles were grouped to treatment delay [surgical delay (n = 1); timing of surgery n = 4)] and complications [presence of factor (n = 1), hyperthermia (n = 1), hypothermia (n = 1), early postoperative stroke (n = 1), pulmonary artery temperature on ICU admission (n = 1)] (Table 2). Of the seven identified factors, we synthesized 1 mechanism from 1 article for its effect on mortality (Table 3).

### Case-mix factors of in-hospital mortality

In total, 36 articles reported on case-mix factors of in-hospital mortality after CABG. Factors in these articles were grouped to sociodemographic factors [sex (n = 4), Native American status (n = 1), Medicaid insurance or uninsured status (n = 1)], health risks [body mass index (n = 3)],

disease characteristics [CAD diffuseness (n = 1)], disease history [prior PCI (n = 3)], comorbidity burden [atrial fibrillation (n = 1), diabetes (n = 1), dialysis-dependent renal failure (n = 2), metabolic syndrome (n = 1), non-dialysis-dependent renal failure (n = 1), peripheral vascular disease (n = 1), peritoneal dialysis (n = 2), preoperative neurological events (n = 1), preoperative reduced ejection fraction (n = 1), QT prolongation (n = 1), renal dysfunction (n = 1), renal insufficiency (n = 1), right ventricular systolic dysfunction (n = 1)], and operative risks [Cockcroft-Gault formula to evaluate renal function (n = 1), C-reactive protein (n = 1), forced expiratory volume 1 (n = 1), left atrial expansion index (n = 1), red cell distribution width (n = 1), REMEMBER score (n = 1), serum creatinine (n = 1), white blood cell count (n = 1)] (Table 2). Of the 27 identified factors, we synthesized 18 mechanisms from 24 articles for their effect on mortality (Table 3).

## Discussion

### Summary of evidence

**Fig. 2:**
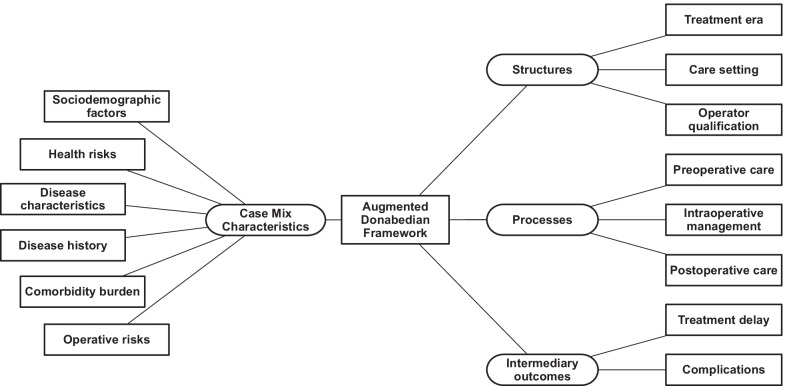
Post-operative mortality factor groups within the augmented Donabedian framework

The purpose of this scoping review was to map factors of in-hospital mortality after CABG to the augmented Donabedian framework of quality improvement, and to synthesize mechanisms for their effect on mortality. We selected factors of mortality reported in 83 articles and sorted them into 14 groups according to common attributes. We mapped the groups to case-mix, structure, process, and outcome elements of the augmented Donabedian framework for quality of care (Fig. 2). The majority (44%) of articles reported on the characteristics of patients, their disease, and their health status. Factors related to care processes were reported in 33% of the articles, and structures 13% of the articles. We synthesized 33 mechanisms for factor association on mortality.

These findings suggest that the patient’s demographic characteristics, their social determinants, health risks, disease characteristics, disease history, comorbidity burden, and operative risks are more frequently assessed risk factors of in-hospital mortality. However, factors in these groups are largely unsuitable for quality improvement given the time available for intervention between surgery and in-hospital death, and therefore may be considered for risk stratification of patients, as suggested by Shroyer [17].

Our results showed process factors of mortality were identified in less than half of the reviewed articles, and structural factors in approximately one in ten reviewed articles. This may be a function of data collection practices, if care process documentation is not translated into records in the cardiac surgery database. Equally, data on structural factors of mortality may not be collected at all if the database is for a single institution or if the registry focus is more epidemiological than one that supports health services research. Therefore, an opportunity may exist for cardiac surgery database managers to incorporate collection of information on both structural and process of care factors into their databases.

An interesting finding of our scoping review was the number of studies reporting on the use of cardiopulmonary bypass strategy—specifically on-pump CABG compared to off-pump CABG—as a factor of mortality, with several papers suggesting mechanisms for the effect [28, 52, 55, 62, 71, 78, 84, 85]. Multiple randomized controlled trials [105–108] have shown no difference in mortality at 30 days between the two approaches, with a five-year extension to the CORONARY trial showing no long-term difference [109]. This may be due to differences in the internal validity of the methodological approaches. For example, observational studies cannot control for unobserved confounding. Alternatively, it may be due to differences in the external validity of the approach whereby observational studies better reflect the entire population versus those that are suitable for enrollment into randomized controlled trials.

Shroyer [110] wrote that outcomes indirectly provide information on potential challenges, and do not identify specific actions to be taken. In response, we extracted and synthesized mechanisms for the effect of 52 factors of mortality from 83 articles, approximately 63% of those reviewed. While these results provide insight into the effect of the factors, it offers limited targets for improvement given only 15 mechanisms were identified for factors mapped to structures and processes of care groups, and modifiable case-mix factors, such as BMI, may not be so during the period between surgery and in-hospital mortality. Thus, initiatives to improve care quality will have limited number of factors and information from which to guide their intervention design.

### Limitations

We did not select studies published prior to 2000 to minimize the potential biasing effect of surgical advancements and changes in delivery of coronary artery bypass grafting [1]. This may have led to an underestimation of the extent of prognostic factors of mortality. We limited our search to works published in English and in PubMed, CINAHL, or EMBASE. Additional studies may be non-English and/or published in databases not included in our search strategy. This may have led to an overestimation of the extent of prognostic factors of mortality as positive results are more likely to be published and reported in English language studies [111]. We excluded randomized controlled trials as their findings do not necessarily reflect mortality after coronary artery bypass grafting following usual care. While this may have led to exclusion of potentially relevant literature, observational data reflects real-world mortality and can better inform quality improvement efforts. We excluded studies that used a composite measure of complications and mortality. We excluded studies which did not complete regression analysis as we used regression effect estimates to enable identification of the direction of the reported association [112]. Further, we limited our search strategy to studies of isolated coronary artery bypass grafting due to different projected outcomes across procedures for coronary revascularization [113]. The results are therefore not generalizable to other revascularization procedures. We also limited our search to mortality in hospital to reduce the likelihood of unobserved factors confounding mortality outcomes after discharge from hospital. With reductions in acute length of stay, it is possible we underestimated the extent of prognostic factors of in-hospital mortality [114]. We used statistical significance to identify the presence of an association between the factor and mortality; this work does not describe the strength of the association which may further inform which factors to target for intervention. When selecting factors, we reported the presence of an association, not the strength of the association. Finally, we did not assess the quality of the reviewed articles per the scoping review framework [115]

## Conclusion

Previous research reports numerous factors of post-operative mortality in patients undergoing CABG. This evidence has not been mapped to the conceptual framework of quality improvement.

We identified 52 factors of mortality reported in 83 articles and mapped them to 14 groups of contributing to mortality onto the augmented Donabedian framework for quality of care, which includes case mix, structure, process, and intermediary outcomes. Most factors included proposed mechanisms for their mortality effects. The majority of factors reported were immutable factors, related to characteristics of patients, their disease and their pre-operative health status. Modifiable factors related to care structures and intermediary outcomes were least reported, with factors related to care processes reported in only one-third of the articles. Therefore, there are limited evidence-based opportunities to improve mortality that will reduce variation in mortality after coronary artery bypass graft surgery. Future studies should consider studying modifiable factors that may be intervened upon to improve mortality directly or through their modifiable mechanism.

## Supplementary Information

**Additional file 1**. **APPENDIX A.** Pubmed search to identify factors of in-hospital mortality. **APPENDIX B.** Cinahl search to identify factors of in-hospital mortality. **APPENDIX C.** Embase (EMBASE.COM) search to identify factors of in-hospital mortality. **APPENDIX D.** Embase (OVID) search to identify factors of in-hospital mortality.

## Data Availability

Data are collected from the published literature; references are detailed in the manuscript.

## Abbreviations

ASA: Acetylsalicylic acid
CABG: Coronary artery bypass graft
CAD: Coronary artery disease
CINAHL: Cumulative index to nursing and allied health literature
EMBASE: Excerpta medica database
ICU: Intensive care unit
PCI: Percutaneous coronary intervention
PRISMA: Preferred reporting items for systematic reviews and meta-analyses
PRISMA-ScR: PRISMA extension for scoping reviews

## References

[1] Head SJ, Kieser TM, Falk V, Huysmans HA, Kappetein AP (2013). Coronary artery bypass grafting: part 1—the evolution over the first 50 years. Eur Heart J.

[2] Loop FD, Lytle BW, Cosgrove DM, Stewart RW, Goormastic M, Williams GW (1986). Influence of the internal-mammary-artery graft on 10-year survival and other cardiac events. N Engl J Med.

[3] Pu A, Ding L, Shin J, Price J, Skarsgard P, Wong DR (2017). Long-term outcomes of multiple arterial coronary artery bypass grafting: a population-based study of patients in British Columbia, Canada. JAMA Cardiol.

[4] Canadian Institute for Health Information. Cardiac care quality indicators report. Ottawa, ON; 2017.

[5] CorHealth Ontario. Report on adult cardiac srugery: isolated coronary artery bypass graft (CABG) surgery, isolated aortic valve replacement (AVR) surgery and combined CABG and AVR surgery October 2011–March 2016. Toronto, ON; 2018.

[6] Krumholz HM, Chen J, Rathore SS, Wang Y, Radford MJ (2003). Regional variation in the treatment and outcomes of myocardial infarction: investigating New England's advantage. Am Heart J.

[7] Quin JA, Sheng S, O'Brien SM, Welke KF, Grover FL, Shroyer AL (2011). Regional variation in patient risk factors and mortality after coronary artery bypass grafting. Ann Thorac Surg.

[8] National Institute for Cardiovascular Outcomes Research. National Adult Cardiac Surgery Audit—Annual Report 2010–2011. London, UK; 2012.

[9] Flather M, Rhee JW, Boothroyd DB, Boersma E, Brooks MM, Carrie D (2012). The effect of age on outcomes of coronary artery bypass surgery compared with balloon angioplasty or bare-metal stent implantation among patients with multivessel coronary disease. A collaborative analysis of individual patient data from 10 randomized trials. J Am Coll Cardiol.

[10] Hochberg MS, Parsonnet V, Gielchinsky I, Hussain SM (1983). Coronary artery bypass grafting in patients with ejection fractions below forty percent. Early and late results in 466 patients. J Thorac Cardiovasc Surg.

[11] Kulier A, Levin J, Moser R, Rumpold-Seitlinger G, Tudor IC, Snyder-Ramos SA (2007). Impact of preoperative anemia on outcome in patients undergoing coronary artery bypass graft surgery. Circulation.

[12] Birkmeyer JD, Siewers AE, Finlayson EV, Stukel TA, Lucas FL, Batista I (2002). Hospital volume and surgical mortality in the United States. N Engl J Med.

[13] Hannan EL, Wu C, Ryan TJ, Bennett E, Culliford AT, Gold JP (2003). Do hospitals and surgeons with higher coronary artery bypass graft surgery volumes still have lower risk-adjusted mortality rates?. Circulation.

[14] Leavitt BJ, O'Connor GT, Olmstead EM, Morton JR, Maloney CT, Dacey LJ (2001). Use of the internal mammary artery graft and in-hospital mortality and other adverse outcomes associated with coronary artery bypass surgery. Circulation.

[15] Campbell NC, Murray E, Darbyshire J, Emery J, Farmer A, Griffiths F (2007). Designing and evaluating complex interventions to improve health care. BMJ.

[16] Donabedian A (1988). The quality of care. How can it be assessed?. JAMA.

[17] Shroyer AL, London MJ, Sethi GK, Marshall G, Grover FL, Hammermeister KE (1995). Relationships between patient-related risk factors, processes, structures, and outcomes of cardiac surgical care. Conceptual models. Med Care.

[18] Tricco AC, Lillie E, Zarin W, O'Brien KK, Colquhoun H, Levac D (2018). PRISMA extension for scoping reviews (PRISMA-ScR): checklist and explanation. Ann Intern Med.

[19] Organization for Economic Cooperation and Development iLibrary. Health risks. 2020. Available from: https://www.oecd-ilibrary.org/social-issues-migration-health/health-risks/indicator-group/english_1c4df204-en.

[20] Rosenbaum PR (2010). Design of observational studies.

[21] Westreich D, Greenland S (2013). The table 2 fallacy: presenting and interpreting confounder and modifier coefficients. Am J Epidemiol.

[22] Alam M, Lee VV, Elayda MA, Shahzad SA, Yang EY, Nambi V (2013). Association of gender with morbidity and mortality after isolated coronary artery bypass grafting. A propensity score matched analysis. Int J Cardiol.

[23] Allareddy V, Allareddy V, Konety BR (2007). Specificity of procedure volume and in-hospital mortality association. Ann Surg.

[24] Atalan N, Fazliogullari O, Kunt AT, Basaran C, Gurer O, Sitilci T (2012). Effect of body mass index on early morbidity and mortality after isolated coronary artery bypass graft surgery. J Cardiothorac Vasc Anesth.

[25] Azarfarin R, Pourafkari L, Parvizi R, Alizadehasl A, Mahmoodian R (2010). Off-pump coronary artery bypass surgery in severe left ventricular dysfunction. Asian Cardiovasc Thorac Ann.

[26] Benedetto U, Lau C, Caputo M, Kim L, Feldman DN, Ohmes LB (2018). Comparison of outcomes for off-pump versus on-pump coronary artery bypass grafting in low-volume and high-volume centers and by low-volume and high-volume surgeons. Am J Cardiol.

[27] Blankstein R, Ward RP, Arnsdorf M, Jones B, Lou YB, Pine M (2005). Female gender is an independent predictor of operative mortality after coronary artery bypass graft surgery: contemporary analysis of 31 Midwestern hospitals. Circulation.

[28] Borgermann J, Hakim K, Renner A, Parsa A, Aboud A, Becker T (2012). Clampless off-pump versus conventional coronary artery revascularization: a propensity score analysis of 788 patients. Circulation.

[29] Bybee KA, Powell BD, Valeti U, Rosales AG, Kopecky SL, Mullany C (2005). Preoperative aspirin therapy is associated with improved postoperative outcomes in patients undergoing coronary artery bypass grafting. Circulation.

[30] Chen S, Li J, Li Q, Qiu Z, Wu X, Chen L (2019). Metabolic syndrome increases operative mortality in patients with impaired left ventricular systolic function who undergo coronary artery bypass grafting: a retrospective observational study. BMC Cardiovasc Disord.

[31] Chikwe J, Castillo JG, Rahmanian PB, Akujuo A, Adams DH, Filsoufi F (2010). The impact of moderate-to-end-stage renal failure on outcomes after coronary artery bypass graft surgery. J Cardiothorac Vasc Anesth.

[32] Connolly TM, White RS, Sastow DL, Gaber-Baylis LK, Turnbull ZA, Rong LQ (2018). The disparities of coronary artery bypass grafting surgery outcomes by insurance status: a retrospective cohort study, 2007–2014. World J Surg.

[33] Dacey LJ, Munoz JJ, Johnson ER, Leavitt BJ, Maloney CT, Morton JR (2000). Effect of preoperative aspirin use on mortality in coronary artery bypass grafting patients. Ann Thorac Surg.

[34] Dacey LJ, DeSimone J, Braxton JH, Leavitt BJ, Lahey SJ, Klemperer JD (2003). Preoperative white blood cell count and mortality and morbidity after coronary artery bypass grafting. Ann Thorac Surg.

[35] Davierwala PM, Leontyev S, Verevkin A, Rastan AJ, Mohr M, Bakhtiary F (2016). Temporal trends in predictors of early and late mortality after emergency coronary artery bypass grafting for cardiogenic shock complicating acute myocardial infarction. Circulation.

[36] Engel AM, McDonough S, Smith JM (2009). Does an obese body mass index affect hospital outcomes after coronary artery bypass graft surgery?. Ann Thorac Surg.

[37] Foroughi M, Karkhaneh Yousefi Z, Majidi Tehrani M, Noori Foroutaghe A, Ghanavati A, Hassantash SA (2009). Prolonged QT interval and coronary artery bypass mortality due to heart failure. Asian Cardiovasc Thorac Ann.

[38] Furnary AP, Gao G, Grunkemeier GL, Wu Y, Zerr KJ, Bookin SO (2003). Continuous insulin infusion reduces mortality in patients with diabetes undergoing coronary artery bypass grafting. J Thorac Cardiovasc Surg.

[39] Gan HL, Zhang JQ, Bo P, Wang SX, Lu CS (2010). Statins decrease adverse outcomes in coronary artery bypass for extensive coronary artery disease as well as left main coronary stenosis. Cardiovasc Ther.

[40] Garcia-Fuster R, Argudo JA, Albarova OG, Sos FH, Lopez SC, Codoner MB (2006). Prognostic value of chronic obstructive pulmonary disease in coronary artery bypass grafting. Eur J Cardio-Thoracic Surg.

[41] Glance LG, Osler TM, Mukamel DB, Dick AW (2007). Effect of complications on mortality after coronary artery bypass grafting surgery: evidence from New York State. J Thorac Cardiovasc Surg.

[42] Gopaldas RR, Overbey DM, Dao TK, Markley JG (2013). The impact of academic calendar cycle on coronary artery bypass outcomes: a comparison of teaching and non-teaching hospitals. J Cardiothorac Surg.

[43] Grieshaber P, Schneider T, Oster L, Orhan C, Roth P, Niemann B (2018). Prophylactic intra-aortic balloon counterpulsation before surgical myocardial revascularization in patients with acute myocardial infarction. Perfusion.

[44] Grossi EA, Bizekis CS, Sharony R, Saunders PC, Galloway AC, Lapietra A (2003). Routine intraoperative transesophageal echocardiography identifies patients with atheromatous aortas: impact on "off-pump" coronary artery bypass and perioperative stroke. J Am Soc Echocardiogr.

[45] Halkos ME, Puskas JD, Lattouf OM, Kilgo P, Guyton RA, Thourani VH. Impact of preoperative neurologic events on outcomes after coronary artery bypass grafting. Ann Thorac Surg. 2008;86(2):504–10; discussion 10. 10.1016/j.athoracsur.2008.04.011 18640324

[46] Hannan EL, Samadashvili Z, Wechsler A, Jordan D, Lahey SJ, Culliford AT (2010). The relationship between perioperative temperature and adverse outcomes after off-pump coronary artery bypass graft surgery. J Thorac Cardiovasc Surg.

[47] He XY, Gao CQ (2019). Peri-operative application of intra-aortic balloon pumping reduced in-hospital mortality of patients with coronary artery disease and left ventricular dysfunction. Chin Med J (Engl).

[48] Huffmyer JL, Mauermann WJ, Thiele RH, Ma JZ, Nemergut EC (2009). Preoperative statin administration is associated with lower mortality and decreased need for postoperative hemodialysis in patients undergoing coronary artery bypass graft surgery. J Cardiothorac Vasc Anesth.

[49] Keeling WB, Kilgo PD, Puskas JD, Halkos ME, Lattouf OM, Guyton RA (2013). Off-pump coronary artery bypass grafting attenuates morbidity and mortality for patients with low and high body mass index. J Thorac Cardiovasc Surg.

[50] Kim LK, Looser P, Swaminathan RV, Minutello RM, Wong SC, Girardi L (2016). Outcomes in patients undergoing coronary artery bypass graft surgery in the United States based on hospital volume, 2007 to 2011. J Thorac Cardiovasc Surg.

[51] Koch CG, Li L, Duncan AI, Mihaljevic T, Cosgrove DM, Loop FD (2006). Morbidity and mortality risk associated with red blood cell and blood-component transfusion in isolated coronary artery bypass grafting. Crit Care Med.

[52] Konety SH, Rosenthal GE, Vaughan-Sarrazin MS (2009). Surgical volume and outcomes of off-pump coronary artery bypass graft surgery: does it matter?. J Thorac Cardiovasc Surg.

[53] Lahtinen J, Biancari F, Ala-Kokko T, Rainio P, Salmela E, Pokela R (2004). Pulmonary artery blood temperature at admission to the intensive care unit is predictive of outcome after on-pump coronary artery bypass surgery. Scand Cardiovasc J.

[54] Lee DC, Oz MC, Weinberg AD, Ting W. Appropriate timing of surgical intervention after transmural acute myocardial infarction. J Thorac Cardiovasc Surg. 2003;125(1):115–9; discussion 9–20. 10.1067/mtc.2003.75 12538993

[55] Li Y, Zheng Z, Hu S (2008). Early and long-term outcomes in the elderly: comparison between off-pump and on-pump techniques in 1191 patients undergoing coronary artery bypass grafting. J Thorac Cardiovasc Surg.

[56] Li HY, Chang CH, Lee CC, Wu VC, Chen DY, Chu PH (2017). Risk analysis of dialysis-dependent patients who underwent coronary artery bypass grafting: effects of dialysis modes on outcomes. Medicine (Baltimore).

[57] Liakopoulos OJ, Kuhn EW, Slottosch I, Thielmann M, Wendt D, Kuhr K (2018). Statin therapy in patients undergoing coronary artery bypass grafting for acute coronary syndrome. Thorac Cardiovasc Surg.

[58] Lin Y, Zheng Z, Li Y, Yuan X, Hou J, Zhang S (2009). Impact of renal dysfunction on long-term survival after isolated coronary artery bypass surgery. Ann Thorac Surg.

[59] Lin T, Hasaniya NW, Krider S, Razzouk A, Wang N, Chiong JR (2010). Mortality reduction with beta-blockers in ischemic cardiomyopathy patients undergoing coronary artery bypass grafting. Congest Heart Fail (Greenwich, Conn).

[60] Liu JY, Birkmeyer NJ, Sanders JH, Morton JR, Henriques HF, Lahey SJ (2000). Risks of morbidity and mortality in dialysis patients undergoing coronary artery bypass surgery. Northern New England Cardiovascular Disease Study Group. Circulation.

[61] Lopez-de-Andres A, Jimenez-Garcia R, Hernandez-Barrera V, Perez-Farinos N, de Miguel-Yanes JM, Mendez-Bailon M (2014). National trends in utilization and outcomes of coronary revascularization procedures among people with and without type 2 diabetes in Spain (2001–2011). Cardiovasc Diabetol.

[62] Mack MJ, Brown P, Houser F, Katz M, Kugelmass A, Simon A (2004). On-pump versus off-pump coronary artery bypass surgery in a matched sample of women: a comparison of outcomes. Circulation.

[63] Magee MJ, Jablonski KA, Stamou SC, Pfister AJ, Dewey TM, Dullum MK, et al. Elimination of cardiopulmonary bypass improves early survival for multivessel coronary artery bypass patients. Ann Thorac Surg. 2002;73(4):1196–202; discussion 202–3. 10.1016/s0003-4975(01)03587-1 11996263

[64] Magovern JA, Moraca RJ, Bailey SH, Dean DA, Simpson KA, Maher TD (2010). Preoperative statin is associated with decreased operative mortality in high risk coronary artery bypass patients. J Cardiothorac Surg.

[65] Malaisrie SC, McCarthy PM, Kruse J, Matsouaka R, Andrei AC, Grau-Sepulveda MV (2018). Burden of preoperative atrial fibrillation in patients undergoing coronary artery bypass grafting. J Thorac Cardiovasc Surg.

[66] Mangano DT (2002). Aspirin and mortality from coronary bypass surgery. N Engl J Med.

[67] Massoudy P, Thielmann M, Lehmann N, Marr A, Kleikamp G, Maleszka A (2009). Impact of prior percutaneous coronary intervention on the outcome of coronary artery bypass surgery: a multicenter analysis. J Thorac Cardiovasc Surg.

[68] McNeely C, Markwell S, Vassileva C (2016). Trends in patient characteristics and outcomes of coronary artery bypass grafting in the 2000 to 2012 medicare population. Ann Thorac Surg.

[69] McNeil M, Buth K, Brydie A, MacLaren A, Baskett R (2007). The impact of diffuseness of coronary artery disease on the outcomes of patients undergoing primary and reoperative coronary artery bypass grafting. Eur J Cardio-Thoracic Surg.

[70] Minakata K, Konishi Y, Matsumoto M, Aota M, Sugimoto A, Nonaka M (2000). Influence of peripheral vascular occlusive disease on the morbidity and mortality of coronary artery bypass grafting. Jpn Circ J.

[71] Mizutani S, Matsuura A, Miyahara K, Eda T, Kawamura A, Yoshioka T (2007). On-pump beating-heart coronary artery bypass: a propensity matched analysis. Ann Thorac Surg.

[72] Mohnle P, Snyder-Ramos SA, Miao Y, Kulier A, Bottiger BW, Levin J (2011). Postoperative red blood cell transfusion and morbid outcome in uncomplicated cardiac surgery patients. Intensive Care Med.

[73] Monteiro P (2006). Impact of early coronary artery bypass graft in an unselected acute coronary syndrome patient population. Circulation.

[74] Najafi M, Goodarzynejad H, Karimi A, Ghiasi A, Soltaninia H, Marzban M (2009). Is preoperative serum creatinine a reliable indicator of outcome in patients undergoing coronary artery bypass surgery?. J Thorac Cardiovasc Surg.

[75] Nallamothu BK, Saint S, Saha S, Fendrick AM, Kelley K, Ramsey SD (2001). Coronary artery bypass grafting in Native Americans: a higher risk of death compared to other ethnic groups?. J Gen Intern Med.

[76] Paone G, Brewer R, Theurer PF, Bell GF, Cogan CM, Prager RL (2012). Preoperative predicted risk does not fully explain the association between red blood cell transfusion and mortality in coronary artery bypass grafting. J Thorac Cardiovasc Surg.

[77] Pouleur AC, Rousseau MF, Ahn SA, Amzulescu M, Demeure F, Meester CD (2016). Right ventricular systolic sysfunction assessed by cardiac magnetic resonance is a strong predictor of cardiovascular death after coronary bypass grafting. Ann Thorac Surg.

[78] Pullan M, Kirmani BH, Conley T, Oo A, Shaw M, McShane J (2015). Should obese patients undergo on- or off-pump coronary artery bypass grafting?. Eur J Cardiothorac Surg.

[79] Ramsey SD, Saint S, Sullivan SD, Dey L, Kelley K, Bowdle A (2000). Clinical and economic effects of pulmonary artery catheterization in nonemergent coronary artery bypass graft surgery. J Cardiothorac Vasc Anesth.

[80] Rathore SS, Epstein AJ, Volpp KG, Krumholz HM (2004). Hospital coronary artery bypass graft surgery volume and patient mortality, 1998–2000. Ann Surg.

[81] Rogers MA, Blumberg N, Saint S, Langa KM, Nallamothu BK (2009). Hospital variation in transfusion and infection after cardiac surgery: a cohort study. BMC Med.

[82] Rosenthal GE, Vaughan Sarrazin M, Hannan EL (2003). In-hospital mortality following coronary artery bypass graft surgery in Veterans Health Administration and private sector hospitals. Med Care.

[83] Schneeweiss S, Seeger JD, Landon J, Walker AM (2008). Aprotinin during coronary-artery bypass grafting and risk of death. N Engl J Med.

[84] Sharony R, Bizekis CS, Kanchuger M, Galloway AC, Saunders PC, Applebaum R (2003). Off-pump coronary artery bypass grafting reduces mortality and stroke in patients with atheromatous aortas: a case control study. Circulation.

[85] Sharony R, Grossi EA, Saunders PC, Galloway AC, Applebaum R, Ribakove GH (2004). Propensity case-matched analysis of off-pump coronary artery bypass grafting in patients with atheromatous aortic disease. J Thorac Cardiovasc Surg.

[86] Sobolev BG, Fradet G, Hayden R, Kuramoto L, Levy AR, FitzGerald MJ (2008). Delay in admission for elective coronary-artery bypass grafting is associated with increased in-hospital mortality. BMC Health Serv Res.

[87] Sobolev BG, Fradet G, Kuramoto L, Rogula B (2012). An observational study to evaluate 2 target times for elective coronary bypass surgery. Med Care.

[88] Swaminathan RV, Feldman DN, Pashun RA, Patil RK, Shah T, Geleris JD (2016). Gender differences in in-hospital outcomes after coronary artery bypass grafting. Am J Cardiol.

[89] Thielmann M, Leyh R, Massoudy P, Neuhauser M, Aleksic I, Kamler M (2006). Prognostic significance of multiple previous percutaneous coronary interventions in patients undergoing elective coronary artery bypass surgery. Circulation.

[90] Thielmann M, Neuhauser M, Knipp S, Kottenberg-Assenmacher E, Marr A, Pizanis N (2007). Prognostic impact of previous percutaneous coronary intervention in patients with diabetes mellitus and triple-vessel disease undergoing coronary artery bypass surgery. J Thorac Cardiovasc Surg.

[91] Tooley JE, Bohl DD, Kulkarni S, Rodriguez-Davalos MI, Mangi A, Mulligan DC (2016). Perioperative outcomes of coronary artery bypass graft in renal transplant recipients in the United States: results from the Nationwide Inpatient Sample. Clin Transplant.

[92] Toumpoulis IK, Anagnostopoulos CE, Chamogeorgakis TP, Angouras DC, Kariou MA, Swistel DG (2008). Impact of early and delayed stroke on in-hospital and long-term mortality after isolated coronary artery bypass grafting. Am J Cardiol.

[93] Wang J, Zheng Z, Yang L, Zhang L, Fan H, Hu S (2012). High-sensitive C-reactive protein predicts outcome after coronary artery bypass. Asian Cardiovasc Thorac Ann.

[94] Wang WH, Hsiao SH, Lin KL, Wu CJ, Kang PL, Chiou KR (2012). Left atrial expansion index for predicting atrial fibrillation and in-hospital mortality after coronary artery bypass graft surgery. Ann Thorac Surg.

[95] Wang L, Yang F, Wang X, Xie H, Fan E, Ogino M (2019). Predicting mortality in patients undergoing VA-ECMO after coronary artery bypass grafting: the REMEMBER score. Crit Care.

[96] Warwick R, Mediratta N, Shaw M, McShane J, Pullan M, Chalmers J (2013). Red cell distribution width and coronary artery bypass surgery. Eur J Cardio-Thoracic Surg.

[97] Weerasinghe A, Hornick P, Smith P, Taylor K, Ratnatunga C (2001). Coronary artery bypass grafting in non-dialysis-dependent mild-to-moderate renal dysfunction. J Thorac Cardiovasc Surg.

[98] Weiss ES, Chang DD, Joyce DL, Nwakanma LU, Yuh DD (2008). Optimal timing of coronary artery bypass after acute myocardial infarction: a review of California discharge data. J Thorac Cardiovasc Surg.

[99] Wen HC, Tang CH, Lin HC, Tsai CS, Chen CS, Li CY (2006). Association between surgeon and hospital volume in coronary artery bypass graft surgery outcomes: a population-based study. Ann Thorac Surg.

[100] Woods SE, Noble G, Smith JM, Hasselfeld K (2003). The influence of gender in patients undergoing coronary artery bypass graft surgery: an eight-year prospective hospitalized cohort study. J Am Coll Surg.

[101] Wu SC, Chien LN, Ng YY, Chu HF, Chen CC (2005). Association of case volume with mortality of chinese patients after coronary artery bypass grafting: Taiwan experience. Circ J.

[102] Yanagawa B, Algarni KD, Yau TM, Rao V, Brister SJ (2012). Improving results for coronary artery bypass graft surgery in the elderly. Eur J Cardio-Thoracic Surg.

[103] Zakeri R, Freemantle N, Barnett V, Lipkin GW, Bonser RS, Graham TR (2005). Relation between mild renal dysfunction and outcomes after coronary artery bypass grafting. Circulation.

[104] Zhong H, David T, Zhang AH, Fang W, Ahmad M, Bargman JM (2009). Coronary artery bypass grafting in patients on maintenance dialysis: is peritoneal dialysis a risk factor of operative mortality?. Int Urol Nephrol.

[105] Shroyer AL, Grover FL, Hattler B, Collins JF, McDonald GO, Kozora E (2009). On-pump versus off-pump coronary-artery bypass surgery. N Engl J Med.

[106] Lamy A, Devereaux PJ, Prabhakaran D, Taggart DP, Hu S, Paolasso E (2012). Off-pump or on-pump coronary-artery bypass grafting at 30 days. N Engl J Med.

[107] Houlind K, Kjeldsen BJ, Madsen SN, Rasmussen BS, Holme SJ, Nielsen PH (2012). On-pump versus off-pump coronary artery bypass surgery in elderly patients: results from the Danish on-pump versus off-pump randomization study. Circulation.

[108] Diegeler A, Borgermann J, Kappert U, Breuer M, Boning A, Ursulescu A (2013). Off-pump versus on-pump coronary-artery bypass grafting in elderly patients. N Engl J Med.

[109] Lamy A, Devereaux PJ, Prabhakaran D, Taggart DP, Hu S, Straka Z (2016). Five-year outcomes after off-pump or on-pump coronary-artery bypass grafting. N Engl J Med.

[110] Shroyer AL, Carr BM, Grover FL, Levy A, Goring S, Gatsonis C, Sobolev B, van Ginneken E, Busse R (2019). Health services information: application of Donabedian’s framework to improve the quality of clinical care. Health services evaluation. Health services research.

[111] Gregoire G, Derderian F, Le Lorier J (1995). Selecting the language of the publications included in a meta-analysis: is there a Tower of Babel bias?. J Clin Epidemiol.

[112] Riley RD, Moons KGM, Snell KIE, Ensor J, Hooft L, Altman DG (2019). A guide to systematic review and meta-analysis of prognostic factor studies. BMJ.

[113] Milojevic M, Head SJ, Parasca CA, Serruys PW, Mohr FW, Morice MC (2016). Causes of death following PCI versus CABG in complex CAD: 5-year follow-up of SYNTAX. J Am Coll Cardiol.

[114] McNeely C, Kwedar K, Markwell S, Vassileva CM (2017). Improving coronary artery bypass grafting readmission outcomes from 2000 to 2012 in the Medicare population. J Thorac Cardiovasc Surg.

[115] Levac D, Colquhoun H, O'Brien KK (2010). Scoping studies: advancing the methodology. Implement Sci.

