# Supplementary material for: Factors affecting mortality after coronary bypass surgery: a scoping review
Source: J Cardiothorac Surg. 2022 Mar 21;17:45. doi: 10.1186/s13019-022-01784-z (PMC8935749; doi:10.1186/s13019-022-01784-z)
Supplement: Supplementary file 1 — Additional file 1. APPENDIX A. Pubmed search to identify factors of in-hospital mortality. APPENDIX B. Cinahl search to identify factors of in-hospital mortality. APPENDIX C. Embase (EMBASE.COM) search to identify factors of in-hospital mortality. APPENDIX D. Embase (OVID) search to identify factors of in-hospital mortality. [file 13019_2022_1784_MOESM1_ESM.docx]

Factors Affecting Mortality After Coronary Bypass Surgery: A Scoping Review

Supplemental Material

Hardiman S,^1^ Villan Villan YF,^2^ Conway, JM,^3^ Sheehan KJ,^4^ Sobolev B.^1^

^1^ School of Population and Public Health, University of British Columbia, Vancouver, Canada

^2^ Hospital Universitario La Paz, Madrid, Spain

^3^ Faculty of Medicine, University of British Columbia, Vancouver, Canada

^4^ Department of Population Health Sciences, School of Population Health and Environmental Sciences, King’s College London, London, UK

**APPENDIX A. PUBMED SEARCH TO IDENTIFY FACTORS OF IN-HOSPITAL MORTALITY**

| Step |  | Search Terms |
| --- | --- | --- |
| 1 | Outcome | "Hospital Mortality"[Mesh] OR "Coronary Artery Bypass/mortality"[Mesh] OR post-operative death [Title/Abstract] OR postoperative death [Title/Abstract] OR in-hospital mortality [Title/Abstract] OR hospital mortality [Title/Abstract] OR in-hospital death [Title/Abstract] |
| 2 | Procedure | "Coronary Artery Bypass"[Mesh] OR Aortocoronary Bypass*[tiab] OR Artery bypass*[tiab] OR CABG [Title/Abstract] OR “bypass graft” [Title/Abstract] OR coronary artery bypass graft* [Title/Abstract] |
| 3 | Analysis | Regression[tiab]OR “Regression Analysis”[Mesh] OR “Logistic Models”[Mesh] OR “Proportional Hazards Models”[Mesh] OR “Multivariate Analysis” [Mesh] OR hazard ratio[tiab] OR proportional hazards[tiab] OR logistic [tiab] OR multivariate [tiab] OR odds ratio [tiab] OR “Odds Ratio” [Mesh] |
| 4 | Study Design | cohort studies [mesh:noexp] OR longitudinal studies[mesh:noexp] OR follow-up studies[mesh:noexp] OR prospective studies[mesh:noexp] OR retrospective studies[mesh:noexp] OR cohort[TIAB] OR longitudinal[TIAB] OR prospective[TIAB] OR retrospective[TIAB] OR “Case-Control Studies”[Mesh:noexp] OR "retrospective studies"[mesh:noexp] OR “Control Groups”[Mesh:noexp] OR (case[TIAB] AND control[TIAB]) OR (cases[TIAB] AND controls[TIAB]) OR (cases[TIAB] AND controlled[TIAB]) OR (case[TIAB] AND comparison*[TIAB]) OR (cases[TIAB] AND comparison*[TIAB]) OR “control group”[TIAB] OR “control groups”[TIAB] OR Cross-Sectional Studies[Mesh:noexp] OR “cross-sectional”[TIAB] OR “prevalence study”[tiab] OR “prevalence studies”[tiab] OR "incidence study"[TIAB] OR "incidence studies"[TIAB] OR "transversal study"[tiab] OR "transversal studies"[tiab] OR “Epidemiologic Studies”[Mesh:noexp] OR "Observational Study" [Publication Type] OR "Observational Study as Topic"[Mesh] OR observational stud*[tiab] NOT randomized |
| 5 | Combined | 1 and 2 and 3 and 4 |

Restrictions: January 1, 2000 – December 31, 2019, Humans, English, Adults +19.

**APPENDIX B. CINAHL SEARCH TO IDENTIFY FACTORS OF IN-HOSPITAL MORTALITY**

| Step |  | Search Terms |
| --- | --- | --- |
| 1 | Outcome | MH Hospital Mortality OR TI hospital N3 mortality OR AB hospital N3 mortality OR TI Hospital Mortality OR AB Hospital Mortality OR TI post-operative death OR AB post-operative death |
| 2 | Procedure | MH Coronary Artery Bypass OR TI CABG OR AB CABG OR TI artery N3 bypass OR AB artery N3 bypass OR TI “coronary artery bypass graft*” OR AB “coronary artery bypass graft*” OR TI aortocoronary N3 bypass OR AB aortocoronary N3 bypass |
| 3 | Analysis | MH “Regression” OR MH “Multivariate Analysis” OR MH “Cox Proportional Hazards Model” OR MH "Logistic Regression" OR MH "Odds Ratio" OR TI multivariate OR AB multivariate OR TI regression OR AB regression OR TI ”hazard ratio*” OR “AB hazard ratio*” OR TI logistic OR AB logistic OR TI “odds ratio*” OR AB “odds ratio*” |
| 4 | Study Design | MH “Prospective Studies” OR MH “Case Control Studies” OR MH “Cross Sectional Studies” OR MH “Retrospective Design” OR “Observational Methods” OR TI “prospective stud*” OR AB “prospective stud*” OR TI case N3 control OR AB case N3 control OR TI cross N3 sectional OR AB cross N3 sectional OR TI retrospective OR AB retrospective OR TI observational OR AB observational |
| 5 | Combined | 1 and 2 and 3 and 4 |

Restrictions: January 1, 2000 – December 31, 2019, English, Human, Adults, excludes Medline.

**APPENDIX C. EMBASE (EMBASE.COM) SEARCH TO IDENTIFY FACTORS OF IN-HOSPITAL MORTALITY)**

| Step |  | Search Terms |
| --- | --- | --- |
| 1 | Outcome | 'surgical mortality'/exp/mj OR ‘hospital mortality’:ti,ab OR ‘post-operative death’:ti,ab |
| 2 | Procedure | ‘coronary artery bypass graft'/exp OR ‘CABG’:ti,ab OR ‘artery bypass’:ti,ab OR ‘coronary artery bypass graft’:ti,ab OR ‘bypass graft’:ti,ab OR ‘aortocoronary bypass’:ti,ab |
| 3 | Analysis | ‘risk’/exp OR 'multivariate logistic regression analysis'/exp OR 'multivariate analysis'/exp OR 'proportional hazards model'/exp OR regression:ti,ab OR hazard ratio:ti,ab OR proportional hazards:ti,ab OR logistic:ti,ab OR multivariate:ti,ab OR odds ratio:ti,ab |
| 4 | Study Design | Cohort:ti,ab OR longitudinal:ti,ab OR prospective:ti,ab OR retrospective:ti,ab OR case-control:ti,ab OR cross-sectional:ti,ab OR epidemiologic:ti,ab OR observational:ti,ab |
| 5 | Combined | 1 and 2 and 3 and 4 |

Restrictions: Adult and Aged (19+), January 1, 2000 – December 31, 2015, Humans, English, Exclude Medline, explosion search box not checked.

**APPENDIX D. EMBASE (OVID) SEARCH TO IDENTIFY FACTORS OF IN-HOSPITAL MORTALITY)**

| Step |  | Search Terms |
| --- | --- | --- |
| 1 | Outcome | exp *surgical mortality/ or hospital mortality.ti,ab. or post-operative death.ti,ab. |
| 2 | Procedure | exp coronary artery bypass graft/ or CABG.ti,ab. or artery bypass.ti,ab. or coronary artery bypass graft.ti,ab. or bypass graft.ti,ab. or aortocoronary bypass.ti,ab. |
| 3 | Analysis | exp risk/ or exp multivariate logistic regression analysis/ or exp multivariate analysis/ or exp proportional hazards model/ or regression.ti,ab. or hazard ratio.ti,ab. or proportional hazards.ti,ab. or logistic.ti,ab. or multivariate.ti,ab. or odds ratio.ti,ab. |
| 4 | Study Design | (Cohort or longitudinal or prospective or retrospective or case-control or cross-sectional or epidemiologic or observational).ti,ab. |
| 5 | Combined | 1 and 2 and 3 and 4 |

Restrictions: Adult and Aged (19+), January 1, 2016 – December 31, 2019, Humans, English, exclude Medline
